# Clustered Regularly Interspaced Short Palindromic Repeats (CRISPR)-Cas Unleashed: Transforming Gene Editing With Breakthroughs, Applications, and Ethical Dilemmas

**DOI:** 10.7759/cureus.95908

**Published:** 2025-11-01

**Authors:** Balasundari Ramesh, Adegbenro O Fakoya

**Affiliations:** 1 Cellular Biology and Anatomy, Louisiana State University Health Sciences Center, Shreveport, USA

**Keywords:** crispr-cas, delivery mechanism, gene editing, knock in, off-targets

## Abstract

The most significant breakthrough in gene editing is the advent of the clustered regularly interspaced short palindromic repeats (CRISPR)-Cas system. This innovative technology enables scientists to insert or delete genes using specific enzymes, facilitating modifications to genomes that can influence an organism's phenotype. The Cas9 enzyme is the most widely used within the CRISPR framework and has already received approval for treating sickle cell disease, with many other applications likely to follow. As this rapidly evolving field continues to advance, it holds great promise for addressing genetic disorders and diseases. This article will explore the various enzymes available in the CRISPR system, the range of diseases and conditions that could be treated using this technology, alternative gene therapy methods, and the ethical considerations surrounding its use.

## Introduction and background

Clustered regularly interspaced short palindromic repeats (CRISPR)-Cas is a groundbreaking gene editing technology that enables precise modifications to DNA within cells [1]. CRISPR was first discovered in *Escherichia coli* by Ishino and colleagues in 1987 [2,3]. A breakthrough came when it was found that CRISPR spacers contained sequences derived from foreign viral DNA. This finding led to the hypothesis that CRISPR could function as a bacterial immune system, enabling bacteria to recognize and defend against viral infections [4]. This system allows scientists to accurately alter genetic sequences, which can be instrumental in treating diseases, preventing their spread, and correcting genetic defects. Initially discovered in bacteria, CRISPR-Cas functions by targeting specific DNA sequences with RNA and using the Cas9 enzyme to create cuts at those sites. The cell's natural repair processes can then be harnessed to disrupt genes or insert new DNA. In recognition of this groundbreaking work on CRISPR-Cas9, Emmanuelle Charpentier and Jennifer Doudna were awarded the Nobel Prize in Chemistry in 2020 [5,6].

While gene-editing techniques have existed for many years, they have often faced challenges, including difficulties in precisely targeting genes and issues related to immune responses. Previous methods, such as zinc finger nucleases (ZFNs) and transcription activator-like effector nucleases (TALENs), were complex, labor-intensive, and time-consuming [7]. The advent of CRISPR-Cas has transformed the landscape of gene editing, offering a more straightforward and adaptable approach that enhances practicality and opens new avenues for advancements in both human and agricultural applications [8].

**Figure 1:**
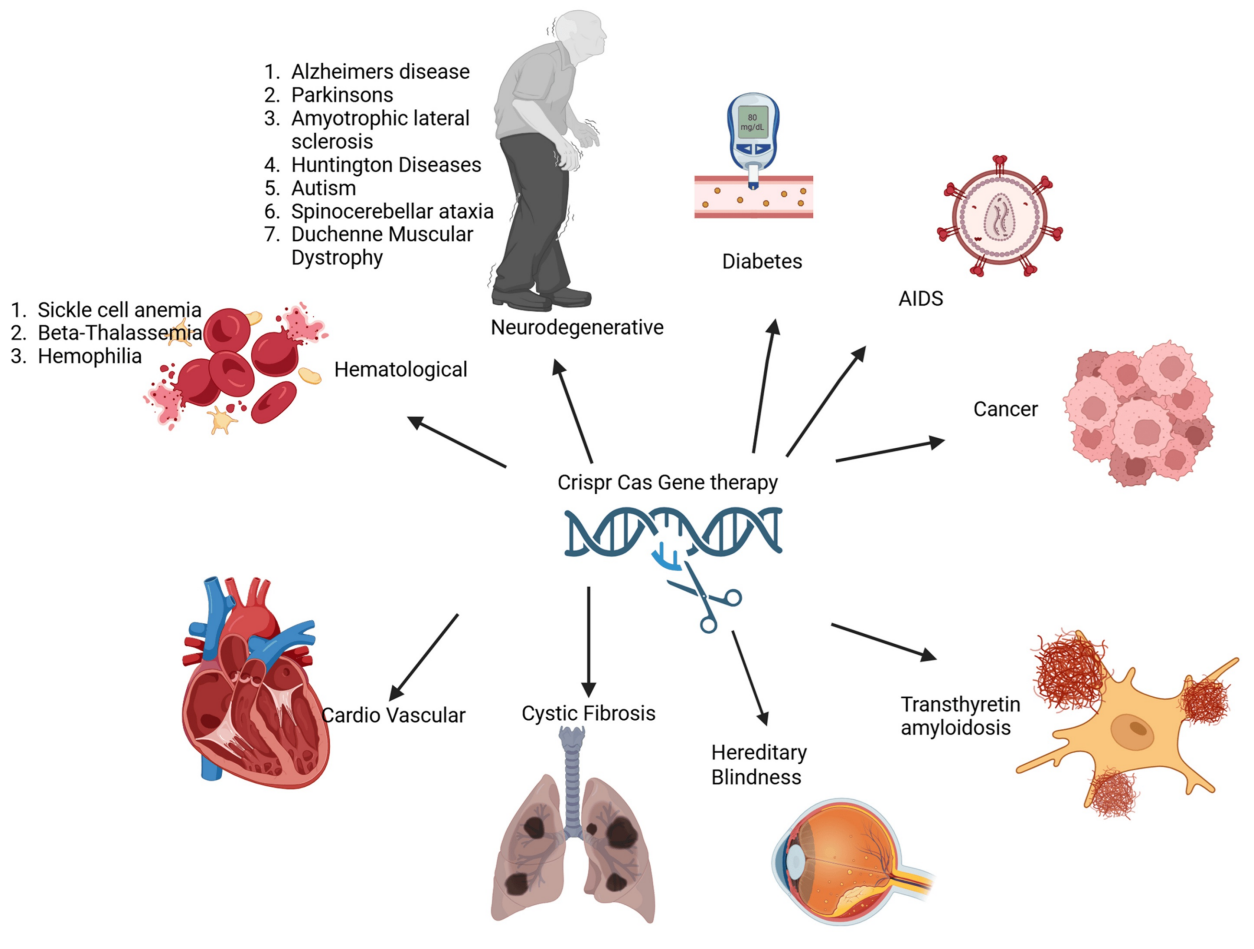
Graphical image of CRISPR-Cas gene therapy to treat various diseases. This figure illustrates the application of CRISPR-Cas gene-editing technology in treating a wide range of genetic and acquired diseases. Disorders, such as sickle cell disease, beta-thalassemia, cystic fibrosis, muscular dystrophy, hereditary blindness, neurodegenerative diseases, and certain cancers, are shown as current targets of CRISPR-based therapies. The figure has been created by Adegbenro O. Fakoya. Created in BioRender (2025). https://BioRender.com/zgvw6hl

In medicine, CRISPR-Cas holds promise for addressing genetic disorders such as sickle cell anemia, Huntington's disease, and infectious diseases such as HIV/AIDS by correcting defective genes. It is also being investigated for cancer therapies, as it can effectively target and disable genes associated with tumor growth (Figure 1). In agriculture, this technology can develop crops that are resistant to diseases and pests, potentially improving yields and nutritional content. Moreover, CRISPR-Cas has the potential to modify insects, such as mosquitoes, to help control diseases like malaria by reducing their ability to transmit the pathogen [9-19].

## Review

Types of CRISPR-Cas

CRISPR systems can be categorized into two main classes based on their structural composition: Class 1 and Class 2. Each class encompasses various types that have distinct mechanisms and components [14].

Class 1 Systems

These systems are characterized by multiprotein complexes and include:

Type I: Type I systems in the interference process are characterized by effector factors, such as the nuclease Cas3 and multiprotein complexes known as Cascade. There are seven distinct subtypes, I-A, I-B, I-C, I-U, I-D, I-E, and I-F, defined by the number of Cascade proteins and the repeat sequences they contain. Typically, Cascade proteins in the Type I system include Cas5, Cas6, Cas7, and Cas8 (Cse1), with some subtypes also containing Cas4 and/or Cas11 (Cse2) [15]. Each subtype has unique Cas proteins and slight differences in the structure of the Cascade complex. An example is the *Escherichia coli* K-12 type I-E system. This system relies on a CRISPR-associated complex (Cascade) to guide the Cas proteins to their target DNA​ [16].

Type III: This type is unique in that it can target both DNA and RNA, leading to RNA degradation in some subtypes. It operates independently of the protospacer adjacent motif (PAM), unlike other types, which identify targets differently. Type III systems use multiprotein complexes composed of various Cas proteins, including the CRISPR-associated multi-subunit RNA-cleaving complexes (Csm and Cmr). These proteins assemble around a guide RNA (crRNA) to form complexes that recognize target sequences [17]. The exact protein composition varies by subtype, with Type III-A and Type III-B being the most well-studied. The complexity and ability of Type III systems to broadly degrade genetic material within a cell pose a challenge, particularly for precisely controlling their activity. While less widely used in genome editing than Cas9, they play an immune response role and carry potential for unintended off-target effects, requiring careful research and ethical consideration, especially when applied in clinical settings [18].

Type IV: Systems are less well-characterized but also fall within the multiprotein classification of Class 1. Unlike other Class 1 systems (such as Types I and III), Type IV CRISPR systems lack some of the typical proteins involved in CRISPR immunity. They do not possess Cas3, a hallmark of Type I systems, nor the typical complex seen in Type III systems. Type IV systems have unique Cas proteins, such as colony-stimulating factor (CSF)1, which is thought to play a central role, along with other distinct proteins, such as CSF2 and CSF3. Due to their complexity and distinct mechanisms, Type IV systems are not currently used in gene editing. However, ongoing research could reveal unique functions that might inspire new biotechnological tools [19].

Class 2 Systems

These systems are simpler and defined by a single effector protein. The notable types include:

Type II: This is perhaps the most well-known due to the popularity of the Cas9 protein, which uses a dual RNA guide (crRNA and tracrRNA) to create double-strand breaks in target DNA. This system has been widely used in genome-editing technologies​ [20]. CRISPR-Cas9 is a powerful gene-editing tool that enables precise alterations to DNA by using guide RNA to direct the Cas9 enzyme to specific genetic locations. This system does not require the engineering of unique protein pairs for each target site; instead, it relies on RNA-DNA base pairing rules to introduce Cas9 to the designated sequence. The implications for gene therapy are substantial, with potential treatments for genetic disorders like cystic fibrosis and muscular dystrophy [21]. Additionally, CRISPR-Cas9 is valuable for disease modeling, allowing researchers to investigate the effects of specific genetic modifications and identify new therapeutic targets [22]. However, it faces challenges, such as off-target effects that can result in unintended genome alterations, as well as ethical concerns, particularly regarding its application in human embryos. Ongoing efforts aim to enhance the precision and efficiency of CRISPR-Cas9, including the development of more accurate Cas9 variants and improved delivery methods. This technology holds promise for broader applications, including treatments for infectious diseases and cancer, as well as agricultural advancements [23].

Type V: Similar to Type II, but it employs a different protein called Cpf1 (also known as Cas12). Cpf1 is guided by a single crRNA and makes staggered DNA cuts, producing "sticky" ends that can be beneficial for specific applications. CRISPR-Cas12a, an alternative to Cas9, is an RNA-guided endonuclease derived from bacterial immune systems. Classified as Class 2 Type V CRISPR, it simplifies the gene-editing process by generating its own guide RNA, eliminating the need for an additional RNA component. This efficiency stems from Cas12a's unique structure and mechanisms [24]. In bacteria and archaea, it functions as a defense mechanism against viruses and other invaders, using the Cas12a protein to cut DNA. Cas12a offers advantages over Cas9, including greater cutting efficiency and the need for only a single RNA guide. Unlike Cas9, which requires assistance from other proteins to process its guide RNA, Cas12a can process its own RNA guides, making it a more straightforward option for gene editing [25].

Type VI: CRISPR Class 2 Type VI systems, characterized by the RNA-targeting enzyme Cas13, are primarily used for antiviral therapies, gene silencing, RNA editing, and diagnostics. Cas13 can degrade viral RNA, making it a potent tool against RNA viruses like COVID-19, while also allowing temporary gene suppression by targeting specific mRNA. Its RNA-editing capabilities enable correction of mutations without altering DNA, and diagnostic platforms such as SHERLOCK use Cas13 for highly sensitive RNA detection [26]. However, Cas13's RNA-targeting nature means its effects are temporary, requiring repeated delivery, which poses challenges for efficient targeting and increases the risk of immune reactions. Additionally, off-target RNA cleavage remains a concern, necessitating further refinement to improve specificity and minimize unintended impacts [27].

Overall, these diverse CRISPR-Cas systems provide significant flexibility and specificity in gene editing and are utilized in various biotechnological and therapeutic applications​.

Diseases

Hematological Disorders

Sickle cell disease: So far, the only CRISPR treatment approved by the FDA is for monogenic sickle cell disease (SCD). SCD is a form of anemia caused by a mutation at the sixth codon of the β-globin (HBB) gene in the adult hemoglobin tetramer, affecting over 100,000 individuals in the United States [28]. Traditionally, treatments for SCD included blood transfusions and preventive therapies, both of which can have serious side effects. Hematopoietic stem cell transplantation has proven curative for severe SCD, but finding a suitable sibling donor is challenging, and there are risks of rejection [29].

CRISPR-Cas9 therapy for SCD, specifically using Casgevy (exagamglogene autotemcel), works by editing a patient's hematopoietic (blood-forming) stem cells. These stem cells are harvested, edited to increase fetal hemoglobin production, a form that can reduce red blood cell sickling, and then reinfused into the patient. Clinical trials for Casgevy have shown that many patients experience significant improvements, with most becoming free from the severe pain crises associated with SCD for extended periods post-treatment​ [30].

Studies have explored the effectiveness of CRISPR-Cas technology in correcting the HBB mutation, thereby increasing fetal hemoglobin (HbF) levels to counteract the effects of the sickle cell mutation. This approach often utilizes a DNA donor template from individuals with SCD. Another treatment avenue involves viral systems, such as adeno-associated viruses (AAVs), which are advantageous due to their low risk of mutagenesis and genotoxicity at the insertion site [31].

This innovative approach was approved in the U.S. in late 2023, marking a historic moment as the first CRISPR-based therapy for SCD. It offers a promising pathway toward curative treatments that rely on a patient's own cells, potentially freeing individuals from lifelong reliance on transfusions and reducing the disease's impact on their quality of life​.

Beta thalassemia: Recent advances in treating beta thalassemia with CRISPR-Cas9 technology mark a significant step forward in providing potentially curative options. In December 2023, the European Medicines Agency (EMA) recommended approval of the first CRISPR-based therapy, Casgevy (also known as exagamglogene autotemcel or exa-cel), for beta thalassemia and sickle cell disease. This therapy, developed by Vertex Pharmaceuticals and CRISPR Therapeutics, uses CRISPR to edit the patient's hematopoietic stem cells, enabling them to produce fetal hemoglobin by altering the B-cell lymphoma/leukemia (BCL11A) gene [32]. This approach reduces the need for frequent blood transfusions. It improves quality of life for patients with transfusion-dependent beta thalassemia (TDT) or severe sickle cell disease (SCD) when traditional stem cell transplantation is not an option.

Casgevy has shown promising results in clinical trials: the majority of beta thalassemia patients in the primary study were transfusion-free for over a year. Due to the severe and chronic nature of these diseases, Casgevy could provide a one-time, potentially curative treatment. However, there are ongoing safety concerns, particularly due to the high-dose chemotherapy required to prepare patients for treatment and the potential for long-term complications, such as clonal hematopoietic diseases or relapse. Rigorous long-term follow-ups are planned to track these risks for up to 15 years [33].

While Casgevy holds much promise, challenges remain around its high cost, which could limit access for many patients. To address this, stakeholders are exploring financing models, such as value-based pricing, to make the therapy more affordable. Nevertheless, CRISPR-based therapies like Casgevy offer a transformative treatment approach that could vastly improve life expectancy and quality of life for patients with beta thalassemia, laying the foundation for future gene-editing therapies for similar genetic disorders.

Hemophilia: Hemophilia is a genetic disorder that impairs blood clotting. CRISPR-based approaches focus on gene editing to correct mutations in the F8 and F9 genes, which encode clotting factors VIII and IX, respectively. By directly editing these genes, researchers can increase the production of the missing clotting factors in patients' liver cells [34].

One approach has been successful in animal models, where CRISPR-Cas9 corrected the mutation in the Coagulation factor IX (FIX) gene associated with hemophilia B, restoring normal blood clotting. Additionally, lipid nanoparticle (LNP) delivery systems are being explored to efficiently deliver CRISPR components to liver cells, offering a non-viral option that may be safer than traditional viral delivery methods [35].

While human clinical trials are still in development, these preclinical studies suggest that CRISPR-based therapies could provide a one-time treatment option, potentially eliminating the need for regular injections and drastically improving the quality of life for those with hemophilia.

Neurodegenerative diseases

CRISPR-Cas technology may offer new treatment options for neurodegenerative diseases, which are a leading cause of death in the United States, claiming over 270,000 lives in 2016 [36]. These diseases can be sporadic or familial, and the latter may be addressed by CRISPR-based genotype modification in patients predisposed to them.

*Alzheimer's Disease (AD)* 

AD, the most well-known neurodegenerative disorder, has seen promising advancements in gene therapy. AD is characterized by the accumulation of tau and amyloid-β (Aβ), forming plaques and tangles in the brain. While most AD cases are sporadic, some result from mutations in specific genes, such as the amyloid precursor protein (APP). Disrupting the APP mutation using CRISPR-Cas9 has shown potential in reducing AD development in mice [37]. To broaden this approach, researchers are investigating ways to target the C-terminus of the APP gene to decrease Aβ production in the brain.

Researchers at the Alzheimer's Association International Conference in 2023 presented a CRISPR/dCas9 technique that effectively reduced APOE ε4 expression by targeting its promoter in human-derived and murine neuronal models [38]. This approach demonstrated reductions of up to 70% in APOE ε4 expression, suggesting the potential to reduce the accumulation of amyloid-beta (Aβ) and associated neurodegeneration​ [39].

Another research focus has been the CRISPR editing of mutations in the Presenilin (PSEN1) gene, linked to familial forms of AD. Studies have shown that disrupting the PSEN1 M146L mutation in fibroblast cells reduces the production of the neurotoxic Aβ42 peptide and restores more typical Aβ42/40 ratios. These findings suggest that targeting the PSEN1 mutation may improve neuronal health and potentially modify disease progression​ [40].

Overall, these advances demonstrate CRISPR-Cas9's potential to precisely target genetic mutations in AD, laying the groundwork for future therapies that address disease mechanisms at the genetic level. However, further research is essential to confirm the efficacy and safety of these therapies in clinical settings.

Parkinson’s Disease (PD)

PD, another neurodegenerative condition, arises from a lack of dopaminergic neurons and the accumulation of neurotoxic deposits in the basal ganglia and substantia nigra, severely affecting motor functions. Like AD, PD can be sporadic or familial, with familial cases accounting for about 10-15% of instances [41]. These familial cases are most likely to be treated with CRISPR-Cas technology, as they stem from genetic mutations. Specific mutations in genes such as alpha-synuclein (SNCA), leucine-rich repeat kinase 2 (LRRK2), and parkin (PARK2) have been linked to an increased risk of developing PD [42]. Researchers are exploring techniques to mitigate the effects of SNCA mutations to prevent harmful alpha-synuclein accumulation and have modified the LRRK2 gene to alter features associated with PD.

One research direction utilizes CRISPR to correct mutations in genes such as PTEN-induced kinase 1 (PINK1) and parkin RBR E3 ubiquitin-protein ligase (PRKN), which play critical roles in mitophagy, the process by which cells eliminate damaged mitochondria. Disruptions in these genes have been associated with early-onset Parkinson's disease and contribute to neuronal death. By restoring or modulating the expression of these genes, CRISPR-based strategies could potentially slow or reverse PD-related neurodegeneration [43].​

Additionally, researchers at the Massachusetts Institute of Technology have developed an adapted version of CRISPR, known as REPAIR, that targets RNA rather than DNA, enabling gene editing without altering the genome. This system can change specific RNA sequences involved in PD pathology, potentially reversing disease symptoms at the protein level. Such techniques represent safer alternatives to DNA editing and may prove especially beneficial for neurodegenerative diseases like Parkinson's​ [41].

As these techniques advance, they hold the potential to develop more effective PD treatments that address both genetic and sporadic forms of the disease. However, further research and trials are essential for safe application in humans.

Amyotrophic Lateral Sclerosis (ALS)

Amyotrophic lateral sclerosis (ALS), also known as Lou Gehrig's disease, is a neurodegenerative disorder that affects motor neurons, leading to muscle weakness, paralysis, and ultimately death. Familial cases are often linked to mutations in the superoxide dismutase (SOD1) or chromosome 9 open reading frame 72 (c9orf72) genes, which harbor hexanucleotide repeat expansions, the most common genetic cause of ALS [44]. This mutation leads to the production of toxic RNA molecules and proteins that contribute to neurodegeneration. Scientists can create disease models of ALS by altering the SOD1 gene. In preclinical studies, researchers have successfully utilized CRISPR/Cas9 to excise the faulty repeat expansions from the c9orf72 gene in mouse models and human cells derived from ALS patients [45]. These interventions reduced the levels of toxic RNA and small proteins without disrupting the expression of the normal C9ORF72 protein. This suggests that CRISPR/Cas9 could halt or slow disease progression by directly targeting its genetic underpinnings​ [46].

Another breakthrough involved the development of a delivery system for the CRISPR/Cas9 components, using adeno-associated virus (AAV) vectors. AAV is recognized for its effectiveness in delivering genetic material to human cells, making it a favorable choice for gene therapy applications​ [47]. Additionally, ongoing research aims to refine these techniques for potential clinical applications, suggesting that with further development, CRISPR-based therapies could significantly improve outcomes for patients with genetic forms of ALS.

Huntington's Disease (HD)

Huntington's disease (HD) is a neurodegenerative disorder caused by a mutation in the huntingtin (HTT) gene, typically involving an expansion of CAG trinucleotide repeats. This results in the production of a toxic protein that damages brain neurons. CRISPR-Cas9 can be programmed to specifically target this expanded repeat, effectively "cutting out" or modifying the faulty gene region. In studies using animal models, CRISPR-Cas9 reduced toxic protein buildup, alleviating symptoms and improving motor function, which are significant for Huntington's disease management [48].

Recent research highlights that CRISPR-Cas9 can directly edit the HTT gene, reducing protein aggregation in neurons and preventing neurodegeneration without affecting viability in mouse models. By deactivating the mutated HTT gene in the striatum, CRISPR treatment has demonstrated potential to address early disease pathology and even reverse some aspects of the disease's neurotoxic effects [49]. While sporadic cases of HD are rare, CRISPR technology has shown promising preclinical results, particularly with the CRISPR-Cas13d system, which targets RNA rather than DNA. Unlike CRISPR-Cas9, which makes permanent DNA cuts, Cas13d selectively degrades mutant mRNA transcripts responsible for the disease. This RNA-targeting approach is significant for neurodegenerative diseases like HD, where DNA repair in neurons is inefficient and precision in targeting is essential to preserve healthy cells [50]. These results have laid the groundwork for potential clinical trials in humans, though such trials are still in early development. Future studies aim to further refine the safety and efficacy of this RNA-targeting CRISPR therapy, which could eventually offer a much-needed treatment option for HD, a condition with no cure.

Autism

CRISPR-Cas technologies have shown potential for treating autism by targeting specific genetic factors associated with the condition. One of the key breakthroughs involves research on autism-linked genes such as chromodomain helicase DNA-binding protein (CHD8) and sodium voltage-gated channel alpha subunit (SCN2A), which play roles in neural development and brain function. Mutations in these genes are often associated with autism spectrum disorder (ASD), seizures, and intellectual disabilities [51].

Recent studies have used CRISPR-based gene activation, which differs from traditional gene editing by increasing gene expression rather than altering the DNA sequence. For example, enhancing CHD8 expression has shown promise in normalizing cell development in lab models with CHD8 mutations, which otherwise lead to neural abnormalities such as larger-than-average head size and overproliferation of neural progenitors. Similarly, increasing SCN2A expression in neurons has improved cellular functions, potentially reversing developmental issues linked to ASD. This technique could lead to more precise, safer interventions but requires further research on its long-term effects and delivery methods for practical application in humans [52].

Another approach explored is epigenetic editing, in which CRISPR is used to modify gene regulatory elements rather than genes themselves. This could be significant for ASD, as many cases involve a combination of genetic and epigenetic changes. While these methods are promising, they raise ethical concerns about altering traits associated with neurodiversity and require rigorous study to ensure safe, ethical applications [52]. Current trials and research are also exploring brain organoid models to confirm these effects and examine the safety and efficacy of gene activation for autism treatment before moving into clinical settings.

Spinocerebellar Ataxias

Spinocerebellar ataxias (SCAs) are a group of inherited neurodegenerative disorders that affect movement coordination and balance. SCAs, particularly types like SCA3 (also known as Machado-Joseph disease), are often caused by genetic mutations involving trinucleotide repeat expansions, which lead to the production of abnormal proteins that harm neuronal cells [53].

One innovative approach involves using CRISPR-Cas9 to precisely target and reduce these expanded repeats in affected genes, such as ATXN3 for SCA3. Researchers have successfully applied CRISPR-Cas9 in cellular and animal models to remove these excessive repeats, demonstrating potential to restore normal protein function and reduce the toxic effects that damage neurons [54]. This method could potentially reverse or alleviate SCA symptoms by directly addressing the underlying genetic cause.

Additionally, a newer CRISPR technique, CRISPR interference (CRISPRi), is being tested to suppress mutant genes associated with various SCAs. CRISPRi enables researchers to reduce the expression of toxic proteins without altering the DNA sequence. This approach has shown success in early preclinical studies, where it improved motor functions and reduced neurodegeneration in animal models of SCA, suggesting a potential pathway for treating these disorders in humans [55,56].

While these treatments are still in early stages of development, their success in laboratory models offers hope for developing effective gene therapies for SCAs, which currently lack curative treatments and are managed primarily through symptom control.

Duchenne Muscular Dystrophy (DMD)

Duchenne muscular dystrophy (DMD) is a genetic disorder characterized by the absence of the dystrophin protein, which is crucial for muscle stability. Traditionally, gene therapies for DMD have faced challenges due to the large size of the dystrophin gene and the immune response triggered by high doses of viral delivery systems. However, several novel approaches aim to overcome these barriers [57]. One breakthrough involves using the CRISPR-Cas3 system, which has successfully restored dystrophin function in stem cells derived from DMD patients. CRISPR-Cas3, unlike the more familiar Cas9, may offer a more effective way to repair genetic errors across larger regions of DNA, providing a powerful tool for restoring normal protein function in cells affected by DMD​ [58].

Another promising method uses a self-complementary adeno-associated viral vector (scAAV) to deliver the CRISPR-Cas9 components. This approach requires lower doses than those of traditional single-stranded vectors, thereby minimizing immune responses while effectively restoring dystrophin production in affected muscle tissues. Preclinical trials in DMD mouse models have shown that scAAV delivery can result in dystrophin expression in up to 95% of muscle cells at significantly reduced doses, improving both muscle function and resilience against damage. Lee et al. developed a CRISPR-AuNPs vehicle for the direct transfer and rapid delivery of Cas9 ribonucleoprotein and donor DNA repair templates to correct gene alterations in a model of Duchenne muscular dystrophy (MDX) mice [59,60].

These innovations are still in experimental stages but hold the potential for new, long-lasting therapies that might eventually halt or even reverse the progression of muscular dystrophy, with human trials anticipated in the near future.

Diabetes 

CRISPR technology has made significant strides in treating both Type 1 and Type 2 diabetes.

For Type 1 diabetes, CRISPR Therapeutics and ViaCyte have collaborated on a groundbreaking approach using gene-edited stem cells, aiming to replace destroyed insulin-producing pancreatic cells. Their therapy, known as VCTX210, is currently in early clinical trials and is designed to evade immune attacks, potentially reducing the need for immunosuppressive drugs. This therapy shows promise in restoring natural insulin production by introducing new functional beta cells, potentially providing a longer-term solution than insulin injections alone [61].

For Type 2 diabetes, CRISPR is being explored to modify genes associated with insulin resistance and metabolic control. Early animal studies have demonstrated that CRISPR can target genes such as leptin and incretins, which regulate metabolism and glucose levels, potentially improving insulin sensitivity and reducing blood sugar levels. One such study used CRISPR to increase incretin levels in mice, resulting in improved blood glucose control and reduced insulin resistance, with few side effects. Although still in the experimental stage, these approaches highlight the potential for CRISPR to eventually offer more sustainable and less invasive treatment options for Type 2 diabetes than traditional therapies [62].

As these CRISPR-based treatments progress through trials, the goal is to provide long-term or even permanent relief from diabetes symptoms, moving closer to a potential cure or at least a substantial improvement in diabetes management for both types.

Cancer

CRISPR/Cas technology is emerging as a promising avenue for cancer treatment and prevention. One of the first significant applications is chimeric antigen receptor (CAR) T-cell therapy, in which scientists engineer T cells using CRISPR to target and destroy cancer cells within the patient's body. This approach is particularly effective against leukemia, lymphoma, and myeloma by using CRISPR to remove immune checkpoint genes, such as PD-1, in T cells [63]. Scientists hope to prevent cancer cells from evading immune attacks. Early trials have demonstrated that such modified T cells remain active against tumors without causing severe side effects.​

Another strategy involves modifying or removing oncogenes and tumor suppressor genes. Oncogenes, such as KRAS, MYC, and EGFR, can promote tumor growth when mutated or overexpressed. CRISPR is used to knock out these mutated genes, reducing cancer cell proliferation. Conversely, tumor suppressor genes such as TP53, BRCA1/2, and PTEN encode proteins that inhibit tumor growth [64]. Scientists can utilize CRISPR to repair or activate these genes, enhancing the body's ability to combat cancer. This approach, however, faces challenges, such as ensuring that only cancerous cells are edited to avoid damaging healthy tissue.​

CRISPR-Cas9 is also used to alter non-cancerous cells in the tumor's surrounding tissues, creating a less favorable environment for cancer growth. This is particularly useful for tackling solid tumors that resist direct immune cell infiltration [65]. Moreover, by disabling genes that cancer cells use to resist therapies, CRISPR may help restore sensitivity to treatments, such as chemotherapy, which is especially valuable when patients have exhausted other options [63].

Clinical trials are ongoing, with early findings from U.S.-based trials indicating that CRISPR-Cas9-modified T cells can be safely administered to patients with specific cancers. However, the effectiveness and long-term safety are still being evaluated. While CRISPR holds enormous potential, researchers continue to address safety challenges, including off-target effects and the complexities of delivering CRISPR to solid tumors​.

HIV

The CRISPR/Cas system offers a promising approach to treating or potentially curing human immunodeficiency virus (HIV). Its precise DNA-editing capabilities enable researchers to target the provirus within infected cells. One of the main challenges in treating HIV/AIDS with CRISPR is the existence of latency reservoirs, where the virus integrates into the host DNA, increasing the risk of resurgence [66].

Studies have shown that CRISPR-Cas9 can disrupt key regions of the HIV genome, particularly the long terminal repeats (LTRs). These LTR regions are essential for viral replication, and by introducing targeted breaks in these sequences, CRISPR can reduce or stop viral gene expression and replication, potentially rendering the virus inactive within infected cells [67].

CRISPR can target HIV latent reservoirs, making them detectable and allowing for reactivation, which can then be eliminated by the immune system or through medication. This strategy is known as Shock and Kill [68]. Alternatively, the Block and Lock strategy aims to prevent the virus from reactivating, keeping it in a dormant state [69]. Another approach involves targeting the CCR5 coreceptor on T cells; individuals with the CCR5-Δ32 mutation are resistant to HIV infection, offering a more natural disease-prevention method. The results suggest that CRISPR could potentially eliminate or reduce HIV's presence in latent reservoirs, where the virus remains hidden from conventional therapies [70].

Since HIV can sometimes escape single-target approaches due to its genetic diversity, combining CRISPR-Cas9 with traditional antiretroviral treatments or designing multi-target CRISPR strategies is under investigation. This combined strategy aims to prevent viral escape and enhance the effectiveness of CRISPR interventions [71].

While these approaches are promising, challenges remain, including the risk of off-target effects and the difficulty of delivering CRISPR tools to all infected cells throughout the body. Clinical trials are ongoing to evaluate the safety and efficacy of CRISPR-Cas9 for HIV treatment, offering hope that it may one day lead to a functional cure for the virus.

Cardiovascular disease (CVD)

Research is also exploring CRISPR applications for cardiovascular disease (CVD), where current treatments mainly focus on lifestyle changes and medications. Researchers are exploring its application across various cardiovascular disorders, including congenital heart disease, hyperlipidemia, and inherited arrhythmias. For instance, targeting the PCSK9 and ANGPTL3 genes may reduce the risk of developing CVD. The PCSK9 gene regulates low-density lipoprotein (LDL) levels, while ANGPTL3 controls triglycerides and cholesterol. Modifying these genes could decrease the likelihood of atherosclerosis, the primary underlying cause of CVD [72].

A gene-editing approach using CRISPR-Cas9 in mouse models has been shown to correct mutations in the PRKAG2 gene, which is associated with Wolf-Parkinson-White syndrome, a disorder that causes abnormal heart rhythms. Such targeted editing has shown success in reversing disease phenotypes and restoring cardiac function in these models [73]. Lebek et al. in 2023 [74] used CRISPR-Cas9 base editing to ablate the oxidative activation sites of CaMKIIδ, a primary driver of cardiac disease. In vitro and in vivo editing of the CaMKIIδ gene in iPSC-derived cardiomyocytes and in mice to eliminate oxidation-sensitive methionine residues confers protection from cardiac ischemia/reperfusion (IR) injury.

Additionally, CRISPR could be used to edit foam cells and macrophages that contribute to plaque buildup by targeting genes like CD36 and SR-A. Other CVDs, such as hypertrophic cardiomyopathy (HCM) and Long QT syndrome, could also be addressed through gene editing. HCM can arise from mutations in the MYBPC3 gene, and correcting this mutation may restore heart muscle strength [75]. For Long QT syndrome, gene mutations in KCNQ1, SCN5A, or KCNE1 can reduce arrhythmia risk [76]. Moreover, CRISPR may facilitate the repair of damaged heart tissue by modifying cardiac stem cells, which could be introduced to injured areas to promote the growth of new heart cells, thereby strengthening the affected heart [77].

CRISPR-based therapies are also under investigation for conditions like ATTR amyloidosis, where toxic protein accumulation leads to heart failure. Early clinical trials have demonstrated that CRISPR can significantly reduce levels of harmful proteins, showing potential as a therapeutic strategy for inherited forms of heart failure [78]. Although promising, CRISPR applications in cardiovascular diseases face challenges, including precise delivery to cardiac tissue and minimizing off-target effects. As clinical trials progress, they will offer further insight into the potential and safety of CRISPR-Cas9 in treating cardiovascular diseases.

Cystic fibrosis

Recent advances in CRISPR-based treatments for cystic fibrosis (CF) have shown promising results, primarily through base-editing approaches targeting the CFTR mutations that cause the disease. Notably, researchers are exploring ways to correct these mutations by utilizing CRISPR-associated base editors, such as cytidine base editors (CBEs), which can convert specific base pairs in DNA with high precision. This technique is beneficial for CF, which results from various mutations in the CFTR gene, including some that produce premature stop codons (nonsense mutations) that halt protein production prematurely [79].

A key development in this field is the creation of "mini-organs," or organoids, derived from CF patient stem cells. These lab-grown organoids mimic lung and gut tissues, offering a testbed to assess CRISPR treatments ex vivo. For instance, researchers have successfully edited CFTR mutations in these organoids, restoring function without significant off-target effects. These mini-organs allow repeated rounds of gene editing to ensure precision, making them a safe platform for testing CRISPR's efficacy before potential patient use. Additionally, this approach bypasses immune response issues often associated with in vivo CRISPR therapies​ [80].

Another advancement is the CRISPR-CGBE (CG base editor) tool, developed to convert a cytosine base to a guanine, directly targeting single-nucleotide mutations with reduced off-target effects. This tool has shown efficient editing in laboratory conditions and provides a foundation for further preclinical trials focused on CF-related mutations​ [81].

While CRISPR treatments for CF are not yet in clinical trials, these approaches represent significant strides toward potentially curative therapies that address the genetic root of CF. Researchers hope that, with further validation, CRISPR-based therapies could enter clinical trials, offering new avenues for patients with mutations that are unresponsive to conventional therapies such as Orkambi [82].

Transthyretin amyloidosis (TTR)

CRISPR-Cas treatments for transthyretin (TTR) amyloidosis aim to target the underlying genetic mutation in the TTR gene, which causes misfolded transthyretin proteins to accumulate in organs like the heart and nerves. The most advanced approach is in vivo gene editing, where CRISPR-Cas9 components are delivered directly to the liver, which is responsible for most TTR protein production, using lipid nanoparticles. A notable clinical trial for this approach is NTLA-2001, conducted by Intellia Therapeutics and Regeneron. In the Phase 1 trial, a single intravenous dose of NTLA-2001 effectively knocked out the mutant TTR gene, reducing circulating TTR protein levels by up to 87% in patients with hereditary TTR amyloidosis with polyneuropathy. This reduction suggests a significant decrease in amyloidogenic TTR production, potentially slowing or halting disease progression [83].

The trial results are promising, as this was the first instance of a CRISPR-based treatment administered directly in the human body, offering a one-time treatment rather than lifelong therapy. However, challenges remain, particularly concerning off-target effects, where unintended genetic changes could occur, posing risks like cancer or autoimmune reactions. Additionally, while lipid nanoparticles improve targeted delivery, there remains a risk of an immune response to the Cas9 protein, which could reduce treatment efficacy or cause inflammation. Longer-term follow-up studies are needed to ensure the sustained safety and effectiveness of these therapies, and researchers continue to refine the precision of CRISPR targeting and delivery mechanisms to mitigate these risks [84].

Hereditary blindness

Recent advances in CRISPR-Cas9 gene editing are showing promising results in treating certain forms of hereditary blindness, particularly in patients with Leber congenital amaurosis (LCA), an inherited retinal disease that causes severe vision impairment from birth. The BRILLIANCE trial, led by Editas Medicine and conducted at multiple clinical sites, including Mass Eye and Ear and the OHSU Casey Eye Institute, tested the CRISPR-based therapy EDIT-101, delivered directly to the retina via injection, to target mutations in the CEP290 gene, which is responsible for some cases of LCA [85].

In the trial, 12 participants received the therapy, and most experienced improvements in visual function and vision-related quality-of-life measures. Key results showed that several patients had measurable gains in visual acuity, including improvements in how they saw and navigated through spaces and how well they recognized objects. Notably, there were no serious adverse events, and reported side effects were mild and resolved over time [86]. This study highlights the feasibility of delivering CRISPR therapies directly to the retina, providing hope for patients with inherited retinal conditions lacking other treatment options.

While these early results are encouraging, the trial's success underlines the need for further studies to optimize the therapy's dose, assess its long-term efficacy, and potentially apply it to younger patients who might benefit even more from early intervention (Tables 1, 2).

**Table 1:** Overview of selected CRISPR-Cas9-based therapeutic approaches in clinical trials. This table summarizes four notable CRISPR-Cas9-based gene-editing treatments under clinical evaluation or recently approved. Each entry includes the treatment name, targeted indication, gene-editing methodology, potential complications, clinical trial status, and supporting reference numbers. Treatments span conditions such as sickle cell disease, cystic fibrosis, transthyretin amyloidosis, and Leber congenital amaurosis (LCA), demonstrating the versatility of CRISPR-Cas9 technology in addressing both systemic and localized genetic disorders. CRISPR: clustered regularly interspaced short palindromic repeats; TTR: transthyretin; BCL11A: B-cell lymphoma/leukemia.

S. no	Treatment name	Indication	Methodology	Complications	Clinical trial details	References
1	Casgevy (Exa-cel)	Sickle cell disease, beta-thalassemia	CRISPR-Cas9 was used to edit the erythroid-specific enhancer of the BCL11A gene in patients' hematopoietic stem cells to increase fetal hemoglobin production.	Off-target effects, immune response to edited cells. High-dose chemotherapy conditioning is needed; risks include potential long-term effects like clonal hematopoiesis and high treatment costs.	FDA approval in 2023; trials demonstrated a significant reduction in vaso-occlusive crises and transfusion needs.	[28-31]
2	PRT101	Cystic fibrosis	CRISPR-based correction of CFTR gene mutations in airway cells.	Possible respiratory complications; off-target edits.	Phase 1 trials in 2024; focused on the safety and efficacy of gene editing in lung tissues.	[79-82]
3	NTLA-2001	Transthyretin amyloidosis	CRISPR-Cas9 was used to knock down the TTR gene in liver cells.	Liver toxicity, potential off-target effects.	Completed Phase 1 trials showing reductions in TTR levels; further studies are ongoing.	[83,84]
4	CRISPR-Cas9 for Leber congenital amaurosis (LCA)	LCA due to RPE65 mutation	In vivo subretinal administration of CRISPR-Cas9 facilitates targeted genome editing of the RPE65 gene within retinal pigment epithelial cells, aiming to restore normal visual function.	Intraocular inflammation, potential vision changes.	Ongoing clinical trials assessing safety and visual function improvements.	[85,86]

**Table 2:** CRISPR-Cas9 therapeutic strategies for genetic diseases: targets and challenges. This table presents an overview of various genetic diseases being targeted using CRISPR-based gene-editing technologies. It outlines the CRISPR method applied, specific gene targets (e.g., HBB, CFTR, APOE), and associated therapeutic goals such as mutation correction, gene function restoration, or protein production reduction. The challenges section highlights key barriers, including off-target effects, delivery efficiency to specific tissues (e.g., HSCs, neurons, retina), immune responses, and ethical concerns. CRISPR: clustered regularly interspaced short palindromic repeats; HTT: huntingtin.

S. no	Disease	CRISPR method	Challenges	References
1	Sickle cell disease	CRISPR-Cas9 knock-in to correct HBB mutations in hematopoietic stem cells (HSCs)	Off-target effects in HSCs; targeted delivery to HSCs; ethical concerns for germline editing if considered	[28-31]
2	Beta-thalassemia	CRISPR-Cas9 knock-in to correct HBB mutations in HSCs	Targeted delivery to HSCs; off-target effects; ethical concerns for potential germline editing in prenatal cases	[32,33]
3	Hemophilia	CRISPR-Cas9 knock-in to correct mutations in F8 (Hemophilia A) or F9 (Hemophilia B) in liver cells	Targeted delivery to liver; risk of immune reactions; ensuring lasting and sufficient clotting factor production	[34-37]
4	Alzheimer's disease	CRISPR-Cas9 knock-down of amyloid-beta production or base editing of risk genes (e.g., APOE)	Targeted delivery to neurons across blood-brain barrier; specificity for target genes; ethical considerations	[39,40]
5	Parkinson's disease	CRISPR-Cas9 knock-down or base editing of LRRK2 or SNCA	Targeted delivery to neuronal tissues; off-target effects; long-term safety concerns	[41-43]
6	Huntington's disease	CRISPR-Cas9 or Cas12a excision of expanded CAG repeats in HTT	Targeted delivery to neurons; specificity in targeting repeats; managing off-target edits in brain tissue	[48-50]
7	Muscular dystrophy (DMD)	CRISPR-Cas9 exon skipping or gene repair to restore dystrophin function	Targeted delivery to muscle tissue; potential immune response; durability and consistency of gene repair	[57-60]
8	Type 1 diabetes	CRISPR-Cas9 knock-in to regenerate insulin-producing pancreatic stem cells	Targeted delivery to pancreas; potential immune response to edited cells; off-target effects	[61,62]
9	Cancer (various types)	CRISPR-Cas9/Cas12a immune cell engineering (CAR-T)	Controlling off-target immune activation; managing tumor heterogeneity; ethical concerns	[63-65]
10	HIV/AIDS	CRISPR-Cas9 knock-out or Cas13 RNA targeting to disrupt viral DNA/RNA in host T-cells	Targeted delivery to infected T-cells; potential for viral resistance; ethical considerations	[66-71]
11	Cystic fibrosis	CRISPR-Cas9 knock-in to repair CFTR mutations in lung epithelial cells	Targeted delivery to lung tissue; achieving high editing efficiency; avoiding immune response	[79-82]
12	Leber congenital amaurosis	In vivo subretinal CRISPR-Cas9 knock-in of RPE65 gene	Targeted delivery to retinal cells; ethical concerns for vision restoration in developing embryos	[85]
13	Hereditary blindness	In vivo subretinal CRISPR-Cas9 knock-in to correct CEP290 mutations	Targeted delivery to retinal tissue; long-term efficacy; possible immune reactions to CRISPR proteins	[86]

Ethical concerns

The application of CRISPR-Cas technology raises several significant ethical concerns. One of the primary issues is determining the extent to which CRISPR should be used, especially regarding human gene editing. The ability to precisely alter DNA prompts moral questions, particularly about the implications of heritable changes that may be passed down to future generations. This has led to calls for stringent regulations and oversight, as discussed at events like the International Summit on Human Gene Editing [87].

Another major concern is ensuring equitable access to CRISPR-Cas technology. If only specific individuals can afford these treatments, the gap between socioeconomic classes could widen, creating new forms of inequality. This highlights the need for policies that make CRISPR therapies accessible to all, preventing disparities in health and well-being [88].

The potential for non-medical enhancements, such as increasing intelligence or improving physical traits like height and muscle mass, also raises ethical questions. Reports from organizations such as the National Academies of Sciences, Engineering, and Medicine (NASEM) advocate using genome editing strictly for severe medical conditions to avoid societal and ethical problems, such as discrimination and challenges to equality [89].

A notable case occurred in 2018 when Chinese biophysicist Jiankui produced genetically altered babies using CRISPR-Cas technology to prevent HIV transmission. The process involved selecting couples based on specific criteria, such as educational background and HIV status. Ultimately, two pregnancies resulted in the birth of twins, Lulu and Nana, but only Nana received the genetic modification intended to protect against HIV [90]. This experiment drew harsh criticism from the scientific and medical communities, raising significant ethical issues around informed consent, the welfare of future generations, and the potential for unforeseen consequences.

Environmental concerns related to CRISPR-Cas also warrant ethical scrutiny. Genetically edited crops might unintentionally harm other species or disrupt ecosystems, such as when crops engineered for pest resistance affect non-target organisms. The risk of these genetic modifications spreading to wild plants raises additional ecological concerns, including the potential reduction in biodiversity [91]. Relying heavily on genetically modified crops could decrease plant variety, making food supplies more vulnerable to pests and diseases. Furthermore, questions arise about who should control genetically modified organisms, as small farmers may face disadvantages if large biotech companies dominate the market, particularly in developing countries.

​These concerns highlight the need for robust ethical frameworks and regulations to guide the development and application of CRISPR technologies in a responsible and equitable manner.

Limitations

Despite its groundbreaking potential, CRISPR-Cas technology faces several notable limitations that impact its effectiveness.

Delivery Mechanisms

Effectively delivering the CRISPR components (Cas9 and the guide RNA) into target cells is a significant hurdle. This delivery must occur in sufficient quantities and at the right time in the cell cycle for editing to be successful. One significant challenge is the use of engineered extracellular vehicles (EVs) for delivering CRISPR-Cas components. While EVs can transport proteins, lipids, and RNA between cells, they struggle to deliver the large mRNA required for CRISPR-Cas9 effectively. A significant issue is the instability of this mRNA, which can degrade quickly within EVs, reducing its efficacy [92]. Additionally, a significant portion of injected EVs tends to accumulate in the liver, rather than reaching the intended target cells. This unintended buildup can lead to decreased treatment effectiveness and potential off-target effects. Current methods for loading CRISPR-Cas9 into EVs are not very effective, and ensuring proper release within target cells remains a challenge [93].

Off-Target Effects

Another major limitation is the propensity for CRISPR/Cas9 to inadvertently target incorrect areas of the genome, potentially causing unintended mutations that could lead to cancer, known as off-target effects (OTEs) [94]. These can complicate the interpretation of results and raise safety concerns for therapeutic applications. Efforts to engineer high-fidelity Cas9 variants aim to reduce these effects, but they still pose a risk​.

The efficiency of CRISPR knock-ins is often low. When attempting to introduce specific changes using homology-directed repair (HDR), which is preferred for knock-ins, the frequency of successful edits can be significantly lower compared to non-homologous end-joining (NHEJ), which is error-prone [95]. NHEJ tends to dominate in many cell types, leading to random insertions and deletions rather than precise edits​.

Immune Response

To evade immune detection, Cas9 can be modified using several strategies to reduce its immunogenicity. One common approach is protein engineering, in which specific epitopes (the portions of Cas9 recognized by the immune system) are altered without compromising the enzyme's function. Additionally, humanizing Cas9 by introducing amino acid changes that make its sequence more similar to human proteins can help minimize immune recognition. Another method involves using smaller Cas9 variants, such as SaCas9 (*Staphylococcus aureus*), which may be less likely to trigger immune responses compared to the widely used SpCas9 (*Streptococcus pyogenes*). Incorporating chemical modifications, such as PEGylation (the attachment of polyethylene glycol molecules), can also shield Cas9 from the immune system. Finally, using immunosuppressive agents alongside Cas9 delivery or encapsulating the enzyme in delivery vehicles, such as lipid nanoparticles, can further help evade immune detection [96-97] (Table 3).

**Table 3:** Strategies to minimize Cas9-induced immune responses in gene editing applications. This table outlines various methods developed to reduce immune recognition and response to CRISPR-Cas9 components during gene editing therapies. Each row describes a specific modification strategy, including protein engineering (e.g., humanized Cas9 and hypoimmunogenic variants), delivery approaches (e.g., mRNA, nanoparticles, viral vectors), and post-editing techniques (e.g., protein depletion). The table compares their efficacy in reducing immune reactions, outlines current limitations such as stability, delivery efficiency, or editing precision, and cites supporting references. These approaches are crucial to improving the safety and longevity of CRISPR-based therapies in clinical applications. CRISPR: clustered regularly interspaced short palindromic repeats; AAV: adeno-associated viruses.

S. no	Modification method	Description	Efficacy	Limitations	References
1	Humanized Cas9 (hCas9)	Modifying bacterial Cas9 (e.g., *Streptococcus pyogenes *Cas9) to resemble human proteins to reduce immune recognition.	Reduces immune response by mimicking human-like epitopes.	Limited studies; potential for immune response against modified protein.	[96]
2	Delivery of Cas9 as mRNA	Using mRNA encoding Cas9 instead of the protein directly, allowing transient expression to minimize immune response.	High efficacy in transient gene expression; reduces lasting immune exposure.	Less stable in vivo; may require repeated dosing for prolonged effect.	[92,93]
3	Encapsulation in nanoparticles	Encapsulating Cas9 protein or mRNA in lipid nanoparticles (LNPs) or polymeric nanoparticles to shield it from immune detection.	Effective in reducing immune detection and enabling targeted delivery.	Delivery efficiency and tissue targeting remain challenging.	[35]
4	Fusion with immunosuppressive proteins	Fusing Cas9 with proteins that suppress immune signaling, such as PD-L1, to reduce immune detection.	Can reduce immune response; promising for in vivo applications.	May impact Cas9 function or efficiency; limited human trials.	[97]
5	Engineering hypoimmunogenic Cas9 variants	Creating mutations in Cas9 to reduce recognition by immune cells without affecting gene-editing efficiency.	Effective in preclinical models; reduces antibody binding.	High development complexity; uncertain long-term safety.	[98]
6	Transient Cas9 delivery using viral vectors	Using transient delivery of Cas9 via viral vectors (AAV, adenovirus) to limit immune exposure.	Allows temporary gene expression; effective in reducing lasting immune response.	Immune response against viral vector; limited to smaller Cas9 variants.	[99]
7	Cas9 protein depletion after editing	Incorporating protein degradation tags (e.g., degrons) to remove Cas9 rapidly after editing.	Reduces duration of immune exposure; effective in transient editing.	Complex to control degradation timing precisely; may reduce editing efficiency.	[100]

Targeting Specific Cells

Delivering CRISPR/Cas9 to the right cells can be difficult, as current delivery methods may be toxic. Researchers are investigating innovative approaches, such as specialized polymers, to improve targeting, particularly of cancer cells. Polymers, such as polyethylenimine (PEI) and poly(lactic-co-glycolic acid) (PLGA), are commonly used to create stable nanoparticles that protect CRISPR components during delivery, facilitate cellular uptake, and enhance endosomal escape, making them promising non-viral delivery vehicles for gene-editing applications [98,99].

Risks of Unintended Edits

Accidental genetic edits can occur during the CRISPR-Cas9 editing process. Successfully delivering CRISPR components into living organisms is fraught with challenges, including the risk of immune reactions and difficulties in targeting specific tissues. Additionally, the Cas9 protein requires a precise DNA sequence to function, which limits its application across different regions of the genome [100,101].

Researchers are continually working to address these limitations, such as optimizing delivery systems, improving HDR efficiency, and developing more specific Cas9 variants to minimize off-target effects. For a more detailed exploration of these limitations and current advancements in the field, you can check out the sources for further reading.

## Conclusions

CRISPR-Cas9 technology has revolutionized the field of genetics and molecular biology, offering unprecedented precision and efficiency in gene editing. Its potential applications are vast, ranging from basic research to therapeutic interventions for genetic disorders, cancer, and other diseases. Key advantages of CRISPR-Cas9 include its ease of use, cost-effectiveness, and the ability to target multiple genes simultaneously, making it a valuable tool for functional genomics and biomedicine. Researchers have made significant strides in using CRISPR to develop treatments for conditions such as sickle cell disease and muscular dystrophy, and ongoing studies continue to explore its applications in agriculture and biotechnology. However, this technology is not without limitations and ethical concerns. Issues such as off-target effects, the efficiency of knock-in strategies, and the challenge of safe and effective delivery systems remain critical hurdles. Moreover, ethical considerations regarding germline editing and potential misuse underscore the need for robust regulatory frameworks to govern its responsible use. While CRISPR-Cas9 offers exciting opportunities for innovation and improvement across many fields, careful consideration of its limitations and ethical implications is essential to ensure its benefits are realized safely and equitably. As research progresses, continued dialogue among scientists, ethicists, and policymakers will be crucial in navigating the complex landscape of gene editing technology.
